# Kinship, dear enemies, and costly combat: The effects of relatedness on territorial overlap and aggression in a cooperative breeder

**DOI:** 10.1002/ece3.8342

**Published:** 2021-11-19

**Authors:** David J. Humphries, Martha J. Nelson‐Flower, Matthew B. V. Bell, Fiona M. Finch, Amanda R. Ridley

**Affiliations:** ^1^ Department of Biological Sciences Macquarie University Sydney New South Wales Australia; ^2^ Pied Babbler Research Project University of Cape Town Rondebosch Western Cape South Africa; ^3^ Department of Biology Langara College Vancouver British Columbia Canada; ^4^ Institute of Evolutionary Biology School of Biological Sciences University of Edinburgh Edinburgh UK; ^5^ DST/NRF Centre of Excellence Percy FitzPatrick Institute for African Ornithology University of Cape Town Rondebosch South Africa; ^6^ School of Biological Sciences University of Western Australia Perth Western Australia Australia

**Keywords:** cooperation, dear enemy, inter‐group interaction, kin selection, kin‐biased overlap, territorial overlap

## Abstract

Many species maintain territories, but the degree of overlap between territories and the level of aggression displayed in territorial conflicts can vary widely, even within species. Greater territorial overlap may occur when neighboring territory holders are close relatives. Animals may also differentiate neighbors from strangers, with more familiar neighbors eliciting less‐aggressive responses during territorial conflicts (the “dear enemy” effect). However, research is lacking in how both kinship and overlap affect territorial conflicts, especially in group‐living species. Here, we investigate kinship, territorial overlap, and territorial conflict in a habituated wild population of group‐living cooperatively breeding birds, the southern pied babbler *Turdoides bicolor*. We find that close kin neighbors are beneficial. Territories overlap more when neighboring groups are close kin, and these larger overlaps with kin confer larger territories (an effect not seen for overlaps with unrelated groups). Overall, territorial conflict is costly, causing significant decreases in body mass, but conflicts with kin are shorter than those conducted with nonkin. Conflicts with more familiar unrelated neighbors are also shorter, indicating these neighbors are “dear enemies.” However, kinship modulates the “dear enemy” effect; even when kin are encountered less frequently, kin elicit less‐aggressive responses, similar to the “dear enemy” effect. Kin selection appears to be a main influence on territorial behavior in this species. Groups derive kin‐selected benefits from decreased conflicts and maintain larger territories when overlapping with kin, though not when overlapping with nonkin. More generally, it is possible that kinship extends the “dear enemy” effect in animal societies.

## Graphical abstract

Fights between neighbors are common in many animal species, and often dictate territorial boundaries. We investigate how relatedness affects territorial overlap and size, and how relatedness and territorial overlap affect the intensity and length of territorial conflicts. We find that having kin as neighbors reduces the length of territorial conflicts and results in larger overlaps and larger territories. Overall, having kin as neighbors confers benefits in this cooperatively breeding bird species.

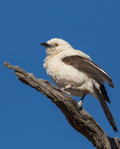

## 1 INTRODUCTION

Territorial defense can help to secure access to resources for breeding or foraging when these resources are limited and in demand (Hinde, 2008). However, there is considerable variation in the extent to which territories are exclusive, and the intensity with which territory holders attempt to repel incursions by others (Christensen & Radford, 2018; Leiser, 2003; Stamps & Buechner, 1985; Temeles, 1994). Any tolerance of territorial overlap or incursion requires explanation, since it is likely to deplete the resources available to the territory holder (Davies & Houston, 1981; Stamps, 1984). The costs of territorial overlap, however, must be balanced against the costs of territorial defense (Low, 2006; Mares et al., 2012). Investment in territorial defense is often dynamic, changing in response to intruder pressure (the number of intruder visits per territory and the level of threat imposed by the intruder), the quality of the resources being defended, and the state of the territory holder (Christensen & Radford, 2018; Davies & Hartley, 1996; Fort & Otter, 2004; Mazerolle & Hobson, 2004; Stamps, 1990).

Understanding and predicting the occurrence and outcome of territorial disputes has given rise to the “dear enemy” and “nasty neighbor” hypotheses (reviewed by Christensen and Radford (2018)). Both of these hypotheses compare the behavioral reaction elicited by known individuals (neighbors) to that elicited by unknown individuals (strangers; reviewed by Christensen and Radford (2018)). The “dear enemy” hypothesis was first proposed by Fisher (1954) and expanded by Getty (1987), to explain cases in which increased familiarity between neighbors leads to decreased levels of conflict. In these cases, neighbors may be more predictable or less likely to usurp the territory holder (Christensen & Radford, 2018). While mainly investigated in species in which individuals or pairs hold territories, this idea has also been supported in group‐living species such as green woodhoopoes *Phoeniculus purpureus* (Radford, 2005), cichlids *Neolamprologus pulcher* (Bruintjes et al., 2016), mountain gorillas *Gorilla beringei beringei* (Mirville et al., 2018a) and weaver ants *Oecophylla smaragdina* (Uy et al., 2019). In contrast, the “nasty neighbor” hypothesis (Müller & Manser, 2007) suggests that more intense conflict arises between neighbors than between strangers, as shown in group‐living species such as banded mongoose *Mungo mungo* (Müller & Manser, 2007), and weaver ants (Newey et al., 2010). Here, neighbors may pose a greater threat than strangers due to larger average size or better condition, or a greater likelihood of usurpation (Christensen & Radford, 2018). Contrasting circumstances (e.g., variation in population density, breeding season vs. nonbreeding season) may cause the same population to exhibit either the “dear enemy” or the “nasty neighbor” response (Jin et al., 2020). Different individuals in groups may also vary in their response depending on their state (e.g., subordinate vs. dominant (Mirville et al., 2018b); reviewed in Christensen & Radford, 2018).

Relatedness is one important factor known to affect the extent of territorial exclusivity. Kin‐biased territorial overlap occurs across a variety of taxa including fish (Griffiths & Armstrong, 2002), birds (Bebbington et al., 2017; Hatchwell et al., 2001), and mammals (Furuichi, 2020; Kitchen et al., 2005; Mirville et al., 2018a; Sera & Gaines, 1994; Støen et al., 2005; Walker et al., 2008). However, both the exact causes and consequences of kin‐biased territorial overlap remain poorly understood. Kin‐biased overlap is likely to represent a form of kin selection, where territory holders are more likely to tolerate the costs of territorial overlap because the beneficiaries are close relatives (Hatchwell, 2010; Kitchen et al., 2005). There is currently very little information about how kinship affects the frequency of territorial disputes between neighbors, and the subsequent investment into territorial defense (Mirville et al., 2018a). It is also currently unclear whether territorial overlap affects the frequency or intensity of territorial disputes between familiar but unrelated neighbors (Bebbington et al., 2017).

Here, we examine the extent of territorial overlap between neighboring groups in relation to kinship, and assess the effects of relatedness and territorial overlap on the intensity of inter‐group interactions (IGIs) in the cooperatively breeding Southern pied babbler, *Turdoides bicolor*. Further, we quantify the costs of territorial defense in this species and investigate the benefits that may arise from having kin in neighboring territories. Southern pied babblers are medium‐sized (75–95 g) group‐living passerines endemic to the semiarid Kalahari Desert (Ridley & Raihani, 2007). Social groups hold year‐round territories that are frequently defended from neighboring groups (on average one IGI for every 4.4 h of observation). These border interactions vary widely in aggression, from purely vocal, ritualized border defenses to physical attacks (Golabek et al., 2012). As with many cooperatively breeding species, average dispersal distances in the southern pied babbler are low and closely related neighboring groups are common within the study population (Nelson‐Flower et al., 2012; Zack, 2010). Here, we use spatial, genetic, and behavioral data from this habituated population to investigate: (1) how kinship affects territorial overlap; (2) the factors (including kinship and territorial overlap) that influence the frequency, duration, and intensity of IGIs; (3) the costs of territorial defense in terms of body mass loss; and (4) the benefits of territorial overlap with relatives in terms of total territory area.

## 2 METHODS

### 2.1 Study site and species

We observed a color‐ringed, habituated population of southern pied babblers at the Kuruman River Reserve, southern Kalahari desert, South Africa (268′58°S, 218′49°E; Ridley & Raihani, 2007). Southern pied babblers live in stable groups consisting of a dominant breeding pair and a variable number of nonreproductive helpers and dependent young. Mean number of adults, birds over 1 year old, was 4.73 ± 1.48 SD (Raihani et al., 2010). The population was observed continuously between 2003 and 2012, with the number of groups observed each year ranging between 12 and 26 (median 18 groups). Groups were visited on average 1.39 times per week, either in the morning (from dawn, average observation time 2.23 h ± 20 min) or in the afternoon (until dusk, average observation time of 1.19 h ± 27 min). Birds were trained to step on an electronic balance (accuracy ±0.1 g) for a mealworm reward, and were weighed at the start and end of every observation session (weighing protocol (Ridley & Raihani, 2007)).

Southern pied babbler IGIs occur frequently and tend to be highly ritualized, predominantly consisting of vocal displays (Golabek & Radford, 2013; Golabek et al., 2012). Displaying groups occupy positions in opposing trees on the territory boundary, taking it in turns to chorus (“choruses” classified as calling bouts involving more than one group member, lasting for more than one second, and with breaks of more than one second between choruses). IGIs can also escalate to active chases and physical fights, though this is rare.

General observations were recorded for each IGI among 16 groups over 8 years (2004–2012), including the location, time, identity of the two groups involved, and whether the IGI escalated to physical fighting. This 8‐year dataset is the basis of our broad‐scale analyses investigating the number of IGIs per breeding season and whether IGIs escalated to fighting. Over the course of a shorter time (2011–2012), detailed observations for IGIs among 12 groups were recorded including the exact time in seconds that each lasted. This 2‐year dataset is the basis of our more fine‐scale analysis examining the duration of individual IGIs, which can vary widely. This dataset was also used to detect longer‐than‐average IGIs, to examine whether longer IGIs are costly in terms of body mass.

### 2.2 Establishing intergroup relatedness

Southern pied babbler groups are typically composed of a dominant breeding pair and their retained offspring; dominant pairs are monogamous, and extra‐pair paternity and maternity is extremely rare (Nelson‐Flower et al., 2011, 2018). Short‐distance dispersal is very common, including to adjoining territories; dominant birds at neighboring groups are often close relatives, and fine‐scale spatial genetic analyses have shown that relatedness between the dominant individuals of neighboring groups provides a useful proxy for relatedness between the groups (Nelson‐Flower et al., 2012, 2018). Dispersal distance is not sex‐biased, leading to similar population‐wide genetic structure between males, between females, and between males and females (Nelson‐Flower et al., 2012). Pedigrees of the population were previously constructed using parentage analysis of genotypes for nine polymorphic microsatellite loci (Nelson‐Flower, Flower, et al., 2018; Nelson‐Flower et al., 2011). We identified two categories of neighboring groups: (i) nonkin: the dominant pairs of neighboring groups are completely unrelated; (ii) kin: at least one dominant individual of one group is closely related (either parents and offspring or siblings) to at least one dominant of the neighboring group. We chose a conservative pedigree‐based approach because calculations of genetic relatedness for full siblings from the microsatellite data were observed to vary widely. No differences have been observed in behavioral reactions to related neighbors that were the same‐sex versus opposite‐sex (Humphries, unpublished data). Overall, 17 of 37 (45.9%) boundaries were shared between kin groups.

### 2.3 Measuring territory size and territorial overlap

We measured the territorial overlap of 37 territory boundaries among 16 groups over 8 years. Group territories were established using 300 GPS points collected during each year (year defined as Sept 1–Aug 31), representing a minimum of 60 h of observation per group per year. If groups were not observed to this threshold, they were not included in that season's territory size calculations, leading to varying sample sizes per year. GPS points were recorded with hand‐held GPS devices, at 15‐min intervals during observation sessions, from the center of the foraging group. Territory sizes were calculated using the “adaptive sphere‐of‐influence local convex hull” (a‐LoCoH) methodology (Getz et al., 2007). A‐LoCoH was performed in R 2.15.2 (R Core Team, 2020) using the “adehabitat” package v. 1.7.2 (Calenge, 2007). Ninety‐five percentage density isopleths were exported from R into ArcGIS 9.3.1 (ESRI, 2009) where territory sizes were measured using the “Hawths tools” extension (Beyer, 2004). The overlap between territories was measured using the “polygon‐in‐polygon” analysis available in Hawths tools and expressed as area in hectares.

### 2.4 Statistical analysis and model selection

Analyses were carried out in R 4.0.3 (R Core Team, 2020) unless otherwise specified; mixed models were constructed using the packages “lme4” v. 1.1.26 (Bates et al., 2015), and “glmmADMB” v. 0.8.3.3 (Skaug et al., 2014). Both continuous and categorical explanatory variables were centered and scaled to standardize these terms (Schielzeth, 2010). This allows direct comparisons of model estimates and effect sizes to be drawn within and between models (Schielzeth, 2010). We created model sets containing combinations of explanatory terms in the R package “MuMiN” v. 1.43.17 (Bartoń, 2016). When sample size was limited, we restricted model sets to those containing the maximum number of terms to avoid over‐fitting. We used Akaike's information criterion adjusted for small sample sizes (AICc; Burnham & Anderson, 2002) to identify the top model set by eliminating all models with ΔAICc > 2 from the best model (the model with the lowest AICc value). The model set was then further reduced by removal of “nested” models (those that are more complicated versions of better‐scoring models (Arnold, 2010)). Model results are presented with the best model(s), the null model, and the full (global) model. Results are presented with estimates and standard errors from the minimal model identified through model comparisons or model averaging. If model averaging was used, 95% confidence intervals are presented; otherwise, *t*‐ or *z*‐values are presented. Estimates and standard errors for terms not found in the minimal models were calculated from the full (global) model. Specific details of response variables and explanatory terms for each analysis are found below.

### 2.5 Territorial overlap

We first investigated variables that could affect territorial overlap between neighboring groups; as explained above, territorial overlap was measured over a breeding season, so the variables investigated were also measured over each breeding season. We used a series of linear mixed models (LMMs); the response variable was territorial overlap area (square‐root transformed to achieve normality). Explanatory terms in the candidate models included whether any dominant birds between the two groups were closely related to one another (kin or nonkin), the difference between the two groups in the mean number of adult birds (>365 days since hatching), and the amount of rainfall across the season (rain is a proxy for food availability; see Wiley & Ridley, 2016). To control for variation in the number of adult birds within a group over the year (as immigration, dispersal, death, and young group members reaching adulthood can affect the group size over time), group size was calculated as a mean for the breeding season (from the number of adult individuals that were present within a group for each observed day of the breeding season). We included the identities of the groups sharing the territory boundary and the breeding season as random terms. The dataset consisted of 37 territory boundaries among 16 groups over eight breeding seasons.

### 2.6 Benefit of territorial overlap

We investigated whether the size and type of territorial overlaps affected the overall territory area of a group over a breeding season. Territory area was set as the response variable in a series of LMMs (area, expressed in hectares, was square‐root transformed to normality). Explanatory terms included the total area of a group's territory overlap with kin groups, the total area of a group's territory overlap with nonkin groups, rainfall over the season and the mean number of adults in the group over the season. Season and group identity were included as random terms. The dataset included 49 territorial areas of 16 groups over 8 years.

### 2.7 Frequency of inter‐group interactions

We examined factors that could affect the number of territorial intergroup interactions (IGIs) over a breeding season. As above, all variables were measured over breeding seasons. The number of IGIs that occurred for each group during the breeding season (September to April) was set as the response variable in a GLMM with a negative binomial distribution. The number of hours of observation per season was included as an offset variable. Explanatory variables included rainfall over the season, the group size difference, relatedness between interacting groups, the area of overlap between territories, and the interaction between relatedness and overlap. Despite relatedness between groups being correlated with area of overlap between groups (see below), we included both terms and their interaction in the global model to understand how these terms affect different components of territorial behavior. Random terms in all models included the combination of the groups sharing the territory boundary and the breeding season. The dataset consisted of the number of IGIs occurring at 37 territory boundaries among 16 groups over eight breeding seasons.

### 2.8 Duration of intergroup interactions

In addition to the frequency of IGIs, we also investigated the duration of IGIs because these can vary widely: groups could encounter one another infrequently, but whether this would result in more‐ or less‐intense IGIs is not clear. During 2011–2012, we recorded the duration of all IGIs observed, defined as the time in seconds from the start of the first chorus produced by the initiating group until half the adults in that group had resumed foraging. We used a GLMM with a negative binomial distribution, with the duration of IGIs (seconds) as the response variable. Explanatory variables included the relatedness between groups, the area of overlap between neighboring territories, the interaction between relatedness and overlap, the group size difference, and the total amount of rainfall (ml) in the fortnight prior to the IGI. No offset variable was included because we stayed with all groups until IGIs concluded. Observation date and the combination of the groups sharing the territory boundary were included as random terms. The dataset included 86 IGIs involving 12 groups on 56 dates.

### 2.9 Escalation following intergroup interactions

IGIs sometimes escalate to physical interactions such as chasing and fighting; such escalations can cause injuries (A.R. Ridley, pers. obs.). To determine the factors that lead to aggression during IGIs, we investigated the proportion of IGIs at each territory boundary that escalated into physical fights or chasing over a breeding season. All variables were calculated over a breeding season. The number of IGIs that escalated, compared to the number of IGIs observed, was set as the response variable in a GLMM with a binomial distribution. We excluded territory boundaries for which we observed no IGIs. Explanatory terms included group size difference, rainfall, area of overlap, whether the groups were kin, and the interaction between kin and overlap. We included the identities of the interacting groups and the season as random terms. The dataset included the number of IGIs that escalated at 33 territory boundaries among 16 groups over eight seasons.

### 2.10 Energetic cost of intergroup interactions

In order to assess whether investment into territorial defense is costly, we investigated the daily weight gain of 30 individuals across two paired sessions. Daily weight gain was established as the number of grams gained per hour between the first recorded weight, collected as the birds came off roost, and a second weight collected at the end of the session on the same morning (at least 1.5 h after the first weight was collected). We compared daily weight gain between days where an IGI had occurred (we set the minimum duration for an IGI at 5 min; this is a conservative choice because average IGI duration is 7m21 s ± 24 s), against a second session where no IGI had taken place. Non‐IGI sessions were chosen because they occurred within the shortest time period from the IGI sessions; paired sessions (IGI and non‐IGI) occurred within 1 week of each other. All paired sessions occurred before breeding began and prior to the arrival of the first rains of the wet season (so that environmental conditions were unlikely to affect the weight differences measured between the two sessions). Data were collected from individuals from 11 different social groups. The difference in weight gain across paired sessions was analyzed using a paired *t*‐test.

## 3 RESULTS

### 3.1 Territorial overlap

**FIGURE 1:**
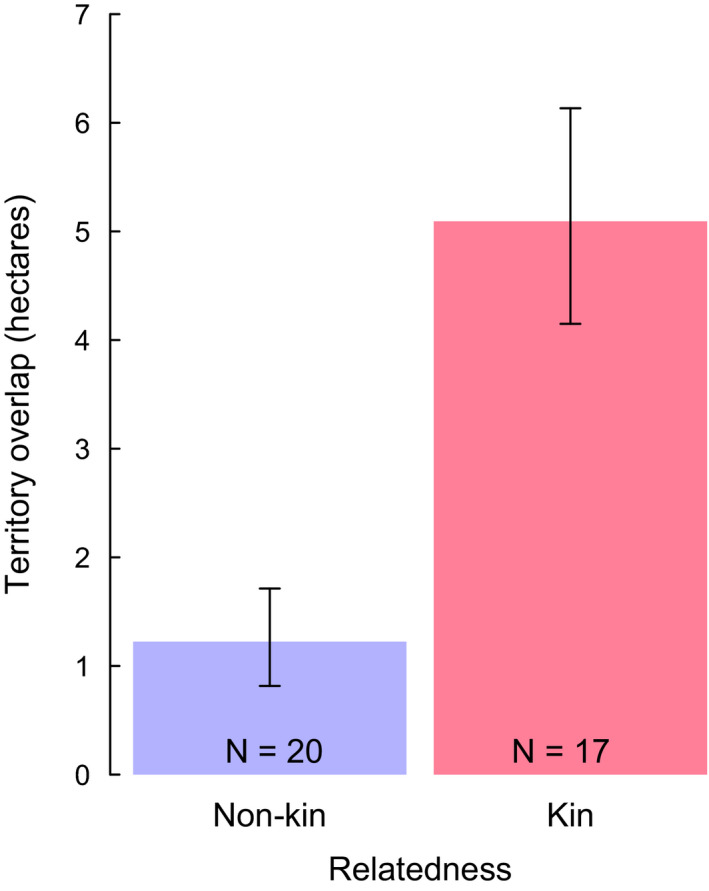
Output from minimal LMM examining territory overlap (hectares) between groups that were nonkin and between groups that were kin. *N* = 37 measured areas of overlap among 16 groups over eight breeding seasons

Neighboring groups shared larger areas of territory when they were closely related (estimate ± standard error: 1.164 ± 0.306; 95% CI: 0.540, 1.788; *p* < .001; Table 1; Figure 1). Overlapping area of kin groups was 6.564 ± 1.565 hectares (*N* = 17 boundaries), representing 9.36% of their average territory size. Nonkin groups shared 1.595 ± 0.313 hectares (*N* = 20 boundaries) representing 2.99% of their average territory size.

**TABLE 1:** Linear mixed models investigating factors affecting area of overlap between neighboring territories

Model	df	Log lik.	AICc	Δ AICc
Relatedness + group size difference	7	−48.92	112.5	0
Relatedness	6	−50.79	114.2	1.7
Full model: relatedness + group size difference + rainfall	8	−49.04	115.6	3.1
Null model	5	−56.51	123.7	11.2

Parameter	Estimate	Standard error	95% CI	*p*
Intercept	1.624	0.158	1.302, 1.946	<.001
Relatedness	1.164	0.306	0.540, 1.788	<.001
Group size difference	−0.489	0.405	−1.296, 0.317	.234
Rainfall^a^	0.149	0.324	0.461^b^	.668

Note

*N* = 37 measured areas of overlap among 16 groups over eight breeding seasons.

^a^ Term estimate, standard error, and *z*‐value determined from the full (global) model.

^b^ *Z*‐value.

### 3.2 Benefit of territorial overlap

**FIGURE 2:**
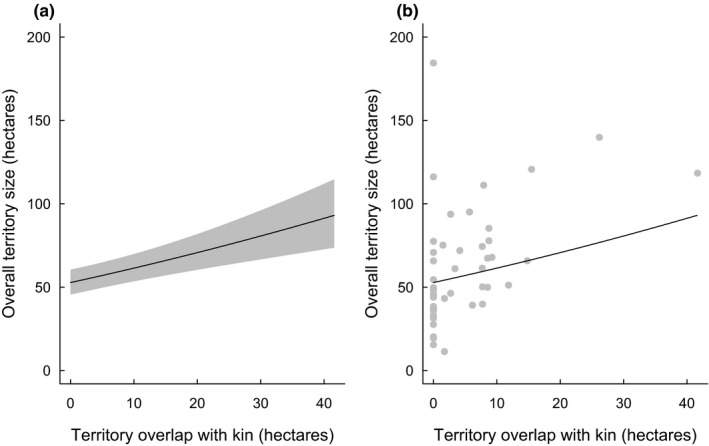
(a) Output from minimal LMM examining territory area overall per size of territory overlap with kin neighbors. (b) Raw data of territory area overall per size of territory overlap with kin neighbors. Regression line from minimal LMM is also shown. *N* = 49 territory area measurements of 16 groups over eight seasons

While larger territory overlaps occurred between neighbors that were kin, it was not clear whether such overlaps translated to territories becoming larger. We found that larger territory overlaps with relatives were associated with greater territory areas (0.878 ± 0.386, *t* = 2.27, *p* = .030, Table 2; Figure 2), but territorial overlap with nonkin had no effect on overall territory size. Mean territory size for groups with larger (than average) overlapping area with kin was 77.384 ± 7.330 hectares, while groups with smaller (than average) overlapping area with kin had territories of 50.965 ± 5.939 hectares. Roughly half of the groups investigated had no measured overlaps with kin (23 of 49 datapoints). Upon inspection, two outlier datapoints had large influences in this analysis: in the first, one group shared territory with two other closely related groups (both related through the dominant female of the focal group: her parents in one and her sister in another). After removal of this datapoint, the larger overlap with kin only tended to be associated with larger territory sizes (0.060 ± 0.034, *t* = 1.76, *p* = .089). A second datapoint had the largest group size area but no territorial overlap with kin; removal of this datapoint resulted in a stronger association of territory size and overlap with kin (0.075 ± 0.026, *t* = 2.91, *p* = .006).

**TABLE 2:** Linear mixed models investigating size of territories

Model	df	Log lik.	AICc	Δ AICc
Overlap with relatives + group size	6	−85.94	186.1	*0*
Full model: overlap with relatives + group size + overlap with nonrelatives + rainfall	8	−84.97	191.2	5.1
Null model	4	−91.52	192.6	6.5

Parameter	Estimate	Standard error	*t* value	*p*
Intercept	7.524	0.503	14.95	<.001
Overlap with relatives	0.878	0.386	2.27	.030
Group size	0.908	0.382	2.38	.023
Overlap with nonrelatives^a^	−0.156	0.392	−0.40	.693
Rainfall^a^	−0.505	0.852	−0.59	.575

Note

*N* = 49 territory area measurements of 16 groups over eight seasons.

^a^ Term estimate, standard error, and *t*‐value determined from the full (global) model.

### 3.3 Frequency of intergroup interactions

IGI frequency was not predicted by group size difference, relatedness between interacting groups, the area of overlap between territories, the interaction between relatedness and overlap, or rainfall (Table 3). IGIs between neighboring groups varied in frequency from 0 IGIs/observation hour (no IGIs observed) to 0.20 IGIs/observation hour, with a mean of 0.05 IGIs/observation hour.

**TABLE 3:** Generalized linear mixed models with negative binomial distributions investigating number of territorial displays per season

Model	df	Log lik.	AICc	Δ AICc
Relatedness	6	−133.24	281.3	0
Overlap	6	−133.70	282.2	0.9
Null model	5	−135.67	283.3	2.0
Full model: group size difference + rainfall + relatedness + overlap + relatedness × overlap	10	−131.07	290.6	9.3

Parameter	Estimate	Standard error	95% CI	*p*
Intercept	−3.191	0.371	−3.948, −2.435	<.001
Overlap	0.594	0.574	−0.546, 1.734	.307
Relatedness	0.242	0.425	−0.603, 1.197	.517
Group size difference^a^	0.097	0.348	0.28^b^	.780
Rainfall^a^	0.711	0.694	1.02^b^	.306
Relatedness × overlap^a^	−2.594	1.942	−1.34^b^	.182

Note

All models included hours observed per season as an offset. *N* = the number of IGIs occurring at 37 territory boundaries among 16 groups over eight breeding seasons.

^a^ Term estimate, standard error and *z*‐value determined from the full (global) model.

^b^ *Z*‐value.

### 3.4 Duration of intergroup interactions

**FIGURE 3:**
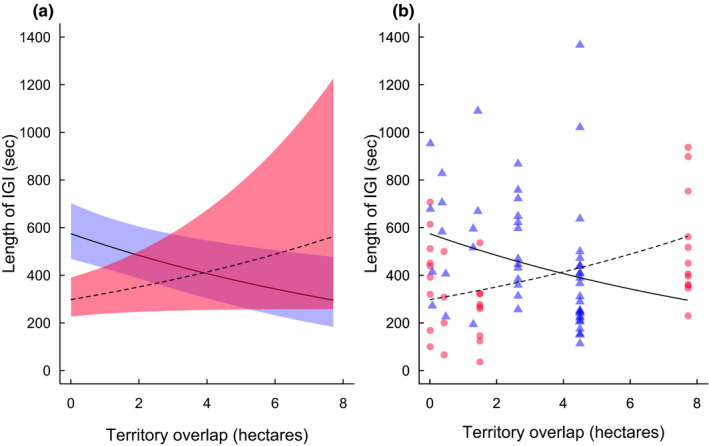
(a) Output from minimal LMM examining duration of IGI per territory overlap (hectares) for nonkin (blue, solid line) and kin (pink, dashed line) neighbors. (b) Raw data of duration of IGIs per territory overlaps for non‐kin (blue triangle) and kin (pink circle) neighbors. Regression lines from minimal LMM (nonkin, solid; kin, dashed) are also shown. *N* = 86 territorial interactions among 12 groups on 56 dates

The duration of individual IGIs was significantly affected by an interaction between relatedness and territorial overlap (0.847 ± 0.324, *z* = 2.62, *p* = .009; Table 4; Figure 3). When opposing groups contained close relatives, IGIs remained similar in duration at all levels of territory overlap (GLMM investigating overlap for related groups: 0.269 ± 0.149, *z* = 1.8, *p* = .071, Figure 3). In contrast, territorial overlap affected length of IGI for nonkin neighbors, such that shorter IGIs occurred with nonkin neighbors that had large overlaps, and longer between nonkin neighbors with small overlaps (GLMM investigating overlap for unrelated groups: −0.563 ± 0.229, *z* = −2.46, *p* = .014, Figure 3). IGIs lasted an average of 391 ± 37 s between kin groups, while between nonkin groups, IGIs lasted an average of 471 ± 37 s.

**TABLE 4:** Generalized linear mixed models with negative binomial distributions investigating factors affecting duration of individual territorial displays

Model	df	Log lik.	AICc	Δ AICc
Relatedness + overlap + relatedness × overlap	8	−580.75	1179.4	0
Null model	5	−586.93	1184.6	5.2
Full model: Relatedness + overlap + relatedness × overlap + rainfall + group size difference	10	−580.63	1184.2	4.8

Parameter	Estimate	Standard error	*z* value	*p*
Intercept	6.033	0.088	68.50	<.001
Relatedness	−0.140	0.159	−0.88	.379
Overlap	−0.098	0.207	−0.47	.637
Relatedness × overlap	0.847	0.324	2.62	.009
Rainfall^a^	0.018	0.122	0.14	.885
Group size difference^a^	0.050	0.112	0.44	.658

Note

*N* = 86 territorial interactions among 12 groups on 56 dates.

^a^ Term estimate and standard error determined from the full (global) model.

### 3.5 Escalation following intergroup interactions

Overall, escalation of territory interactions occurred in 12.5% of IGIs (fighting: 9.7%; chasing: 2.8%). IGI escalation to physical aggression was not predicted by group size difference, relatedness between interacting groups, the area of overlap between territories, the interaction between relatedness and overlap, or rainfall (Table 5).

**TABLE 5:** Generalized linear mixed models with binomial distributions investigating likelihood of observed territorial displays escalating to physical fights or chasing per season

Model	df	Log lik.	AICc	Δ AICc
Relatedness	5	−47.69	107.6	0
Null model	4	−50.05	109.5	1.9
Full model: Relatedness + rainfall + group size difference + overlap + relatedness + overlap × relatedness	9	−46.68	119.2	11.6

Parameter	Estimate	Standard error	95% CI	*p*
Intercept	−2.158	0.338	−2.849, −1.467	<.001
Relatedness	−0.584	0.512	−1.611, 0.444	.265
Rainfall^a^	−0.555	0.517	−1.07^b^	.283
Group size difference^a^	−0.396	0.371	−1.07^b^	.285
Overlap^a^	−0.425	1.044	−0.41^b^	.684
Relatedness × overlap^a^	0.547	1.924	0.28^b^	.776

Note

*N* = 33 counts of territorial displays among 16 groups over eight seasons.

^a^ Term estimate, standard error and *z*‐value determined from the full (global) model.

^b^ *Z*‐value shown here.

### 3.6 Energetic cost of intergroup interactions

**FIGURE 4:**
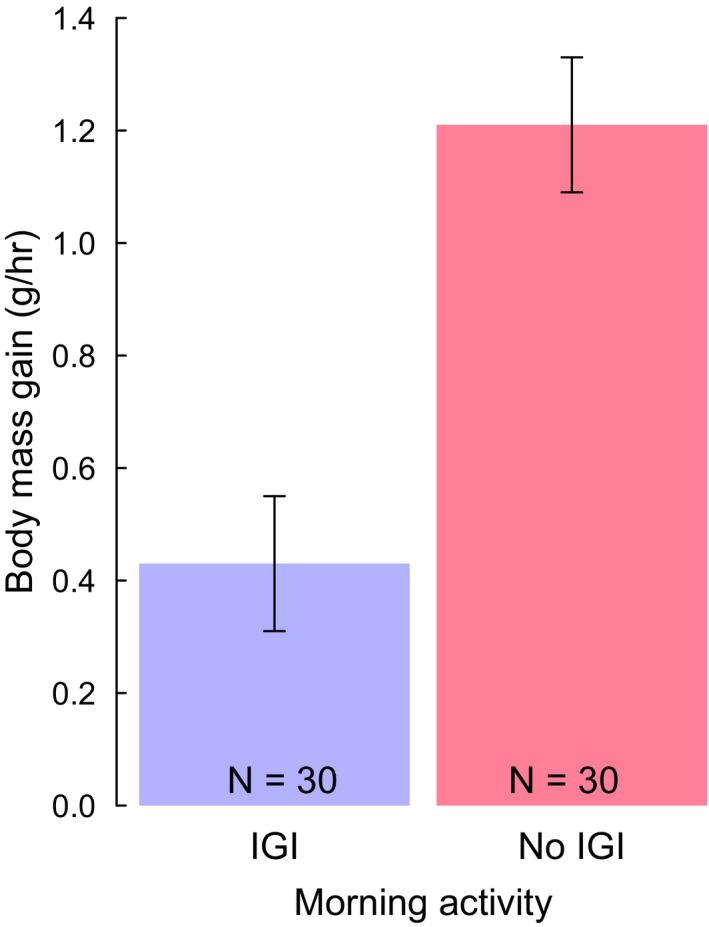
The average hourly weight gain for adults in relation to territorial defense activities across a morning. “No IGI” represents mornings where no intergroup interaction occurred and “IGI” represents mornings when an intergroup interaction occurred

Territorial defense was costly: individuals gained significantly less body mass per hour across mornings when there was at least one IGI compared to mornings when they did not invest in territorial defense behavior (paired *t*‐test, *t* = −4.700, df = 29, *p* < .001, Figure 4). Individuals that engaged in IGI behavior gained 0.43 ± 0.12 g per hour, while those that did not engage in an IGI gained 1.21 ± 0.12 g per hour.

## 4 DISCUSSION

In southern pied babblers, neighboring groups that are more closely related have a greater degree of territorial overlap; groups that have larger overlaps with kin have a larger overall territory than do groups with small overlaps with kin. Taken together, these results suggest that overlaps with related neighbors allow groups to hold larger territories, and that kin selection appears to play a role in territory division. Many group had close relatives living in territories next door to their natal groups; this results from the short dispersal distances for both sexes in this species, and leads to fine‐scale genetic structure detectable for both males and females (Nelson‐Flower et al., 2012; Nelson‐Flower, Wiley, et al., 2018). Species holding year‐round, stable territories may benefit from short‐distance dispersal when kin‐biased territory overlap occurs as a result. Kin‐biased territorial overlap is seen in a number of other species (Griffiths & Armstrong, 2002; Hatchwell et al., 2001; Kitchen et al., 2005; Mirville et al., 2018a; Sera & Gaines, 1994; Støen et al., 2005; Walker et al., 2008). For example, in cooperatively breeding long‐tailed tits *Aegithalos caudatus*, flocks containing related birds had larger overlapping territories; non‐kin flocks that overlapped avoided areas of overlap but kin flocks showed no such avoidance (Hatchwell et al., 2001). Similarly, swift fox *Vulpes velox* are more likely to have overlapping territories when related to one another (Kitchen et al., 2005).

Kin selection appears to play a role in territory division in this cooperatively breeding species. Living near relatives confers benefits: groups living beside relatives have larger overlapping areas and larger territories. Groups perform costly IGIs with kin as often as they do with nonkin neighbors (despite larger territory overlaps with kin), but the interactions are shorter than those with nonkin neighbors. Kin selection theory predicts that closely related individuals should be less aggressive toward one another in agonistic competition. For example, IGIs in mountain gorillas, *Gorilla beringei beringei*, are more likely to be peaceful rather than aggressive when groups contain familiar relatives (Mirville et al., 2018a). Similarly, in Seychelles warblers, *Acrocephalus sechellensis*, males living alongside related males physically fought less often, gained more body mass, and showed less telomere attrition than did those living near nonkin (Bebbington et al., 2017). This effect is also seen in parasitic wasps *Copidosoma floridanum* (Giron et al., 2004). Kin selection also predicts that kin‐biased territory overlap should improve fitness. This is seen in bank voles, *Clethrionomys glareolus*, where females living near their female kin had larger, overlapping territories and experienced significantly better reproductive success (Mappes et al., 1995). Living near (and sharing territory with) related neighbors is likely to bestow net benefits, possibly stemming from either improved foraging or breeding success. Future work investigating the foraging or breeding benefits of larger territories would be useful in clarifying the fitness effects of kin‐biased territory overlap in this species.

Our results show that as nonkin neighbors increase overlap, their investment in IGIs appeared to decrease, a phenomenon known as the “dear enemy” effect (Getty, 1987). This effect occurs as neighbors become more familiar with one another, and results in decreased investment in costly territorial disputes (Christensen & Radford, 2018). In southern pied babblers, we find evidence for this effect between nonkin neighbors when they have a high degree of overlap. However, our results indicate that related groups are already “dear enemies” no matter how much overlap exists between their territories. Here, relatedness confers the benefits of familiarity which can only otherwise be gained with repeated interactions over long periods. This is an important avenue of investigation because most research into “dear enemy” or “nasty neighbor” effects do not take relatedness between groups into account (Bebbington et al., 2017).

While we have interpreted our results regarding IGI durations and territorial overlap in nonkin as supporting the “dear enemy” hypothesis, we must acknowledge that ours is just one interpretation of these results. We found that non‐kin groups with larger territorial overlaps had shorter IGIs, and increased familiarity could be one reason for this result. However, individual differences in dominant pair aggression may play a role in IGI duration and this may also affect the degree of overlap each group is willing to tolerate. We included group identity as a random term in the models to help to control for this type of effect.

Interactions between groups are not necessarily always about displacing neighbors. Encounters between neighboring groups may also play a role in the transfer of information between groups. In black howler monkeys, food availability affects the likelihood that groups approach calling neighbors (Van Belle & Estrada, 2020). Conversely, in brown jays *Cyanocorax morio*, individuals are thought to use interactions as a way of gathering information about potential breeding opportunities (Hale et al., 2003). Similarly, adult subordinate southern pied babblers target loud calling displays toward groups with potential mates (Humphries et al., 2015). Indeed, many southern pied babblers find breeding positions in neighboring groups (Nelson‐Flower et al., 2012). Therefore, displays at territory boundaries may serve to dispute or reinforce territory boundaries while also allowing individuals the opportunity to assess neighboring groups for breeding opportunities or to gain information about resource availability.

The habituation of the southern pied babbler population allowed us to conclusively demonstrate the costs of IGIs, with birds gaining less weight during observation sessions where an IGI had occurred. While most territorial displays involved only loud and prolonged choruses, some IGIs did escalate to chasing and fighting which are likely energetically even more costly and involve the possibility of injury. The costs of territorial defense have previously been measured in several species, both from time‐energy budgets and by assessing the impact of territorial defense on daily weight gain or individual mortality (Davies, 1976; Gill & Wolf, 1975; Low, 2006; Mares et al., 2012; Thompson et al., 2017).

## 5 CONCLUSION

We find that southern pied babblers display kin‐biased territory overlap, and that kinship alters the nature of interactions between neighboring groups. Groups with increased overlap with kin have larger territories overall, which is likely to increase fitness. When interacting with kin at territory boundaries, groups have shorter IGIs; important because engagement in IGIs affect body mass and is costly. Overall, kin selection appears to be a main influence on territorial behavior in this species. Groups are more tolerant of territorial overlap with relatives and derive kin‐selected benefits from shared resources in larger territories. More generally, it is possible that kinship extends the “dear enemy” effect in animal societies.

## CONFLICT OF INTEREST

The authors declare no conflict of interest.

## AUTHOR CONTRIBUTION

**David J. Humphries:** Conceptualization (equal); Data curation (equal); Formal analysis (supporting); Investigation (lead); Methodology (equal); Writing‐original draft (lead); Writing‐review & editing (supporting). **Martha J. Nelson‐Flower:** Conceptualization (supporting); Data curation (supporting); Formal analysis (lead); Investigation (supporting); Writing‐review & editing (lead). **Matt Bell:** Conceptualization (supporting); Investigation (supporting); Methodology (supporting); Supervision (supporting); Writing‐original draft (supporting). **Fiona M. Finch:** Data curation (supporting); Investigation (equal); Writing‐original draft (supporting). **Amanda Ridley:** Conceptualization (equal); Data curation (supporting); Funding acquisition (lead); Investigation (supporting); Methodology (equal); Project administration (lead); Resources (lead); Supervision (lead); Writing‐original draft (supporting); Writing‐review & editing (supporting).

## Data Availability

Data have been deposited in Dryad and are available at https://doi.org/10.5061/dryad.v6wwpzgx3.

## References

[ece38342-bib-0001] Arnold, T. W. (2010). Uninformative parameters and model selection using Akaike’s Information Criterion. The Journal of Wildlife Management, 74, 1175–1178. 10.1111/j.1937-2817.2010.tb01236.x

[ece38342-bib-0002] Bartoń, K. (2016). MuMIn: multi‐model inference.

[ece38342-bib-0003] Bates, D. , Machler, M. , Bolker, B. , & Walker, S. (2015). Fitting linear mixed‐effects models using lme4. Journal of Statistical Software, 67, 1–48. 10.18637/jss.v067.i01

[ece38342-bib-0004] Bebbington, K. , Kingma, S. A. , Fairfield, E. A. , Dugdale, H. L. , Komdeur, J. , Spurgin, L. G. , & Richardson, D. S. (2017). Kinship and familiarity mitigate costs of social conflict between Seychelles warbler neighbors. Proceedings of the National Academy of Sciences, 114, E9036–E9045. 10.1073/pnas.1704350114 PMC5664493 29073100

[ece38342-bib-0005] Beyer, H. L. (2004). Hawth’s Analysis Tools for ArcGIS.

[ece38342-bib-0006] Bruintjes, R. , Lynton‐Jenkins, J. , Jones, J. W. , & Radford, A. N. (2016). Out‐group threat promotes within‐group affiliation in a cooperative fish. American Naturalist, 187, 274–282. 10.1086/684411 26807753

[ece38342-bib-0007] Burnham, K. P. , & Anderson, D. R. (2002). Model selection and multimodel inference, 2nd ed. Springer.

[ece38342-bib-0008] Calenge, C. (2007). Exploring habitat selection by wildlife with adehabitat. Journal of Statistical Software, 22(6), 1–19. 10.18637/jss.v022.i06

[ece38342-bib-0009] Christensen, C. , & Radford, A. N. (2018). Dear enemies or nasty neighbors? Causes and consequences of variation in the responses of group‐living species to territorial intrusions. Behavioral Ecology, 29, 1004–1013. 10.1093/beheco/ary010

[ece38342-bib-0010] Davies, N. B. (1976). Food, flocking and territorial behaviour of the pied wagtail (Motacilla alba yarrellii Gould) in winter. Journal of Animal Ecology, 45, 235. 10.2307/3777

[ece38342-bib-0011] Davies, N. B. , & Hartley, I. R. (1996). Food patchiness, territory overlap and social systems: An experiment with dunnocks Prunella modularis. Journal of Animal Ecology, 65, 837. 10.2307/5681

[ece38342-bib-0012] Davies, N. B. , & Houston, A. I. (1981). Owners and satellites: The economics of territory defence in the pied wagtail, Motacilla Alba. The Journal of Animal Ecology, 50, 157. 10.2307/4038

[ece38342-bib-0013] Fisher, J. (1954). Evolution and bird sociality. In J. Huxley , A. Hardy , & E. Ford (Eds.), Evolution as a process (pp. 71–83). Allen & Unwin.

[ece38342-bib-0014] Fort, K. T. , & Otter, K. A. (2004). Territorial breakdown of black‐capped chickadees, *Poecile atricapillus*, in disturbed habitats? Animal Behavior, 68, 407–415. 10.1016/j.anbehav.2003.08.023

[ece38342-bib-0015] Furuichi, T. (2020). Variation in intergroup relationships among species and among and within local populations of African apes. International Journal of Primatology, 41, 203–223. 10.1007/s10764-020-00134-x

[ece38342-bib-0016] Getty, T. (1987). Dear enemies and the prisoner’s dilemma: Why should territorial neighbors form defensive coalitions? American Zoologist, 27, 327–336. 10.1093/icb/27.2.327

[ece38342-bib-0017] Getz, W. M. , Fortmann‐Roe, S. , Cross, P. C. , Lyons, A. J. , Ryan, S. J. , & Wilmers, C. C. (2007). LoCoH: Nonparameteric kernel methods for constructing home ranges and utilization distributions. PLoS One, 2, e207. 10.1371/journal.pone.0000207 17299587 PMC1797616

[ece38342-bib-0018] Gill, F. B. , & Wolf, L. L. (1975). Economics of feeding territoriality in the golden‐winged sunbird. Ecology, 56, 333–345. 10.2307/1934964

[ece38342-bib-0019] Giron, D. , Dunn, D. W. , Hardy, I. C. W. , & Strand, M. R. (2004). Aggression by polyembryonic wasp soldiers correlates with kinship but not resource competition. Nature, 430, 676–679. 10.1038/nature02721 15295600

[ece38342-bib-0020] Golabek, K. A. , & Radford, A. N. (2013). Chorus‐call classification in the southern pied babbler: Multiple call types given in overlapping contexts. Behaviour, 150, 691–712. 10.1163/1568539X-00003074

[ece38342-bib-0021] Golabek, K. A. , Ridley, A. R. , & Radford, A. N. (2012). Food availability affects strength of seasonal territorial behaviour in a cooperatively breeding bird. Animal Behavior, 83, 613–619. 10.1016/j.anbehav.2011.11.034

[ece38342-bib-0022] Griffiths, S. W. , & Armstrong, J. D. (2002). Kin‐biased territory overlap and food sharing among Atlantic salmon juveniles. Journal of Animal Ecology, 71, 480–486. 10.1046/j.1365-2656.2002.00614.x

[ece38342-bib-0023] Hale, A. M. , Williams, D. A. , & Rabenold, K. N. (2003). Territoriality and neighbor assessment in brown jays (*Cyanocorax morio*) in Costa Rica. The Auk, 120, 446–456. 10.1093/auk/120.2.446

[ece38342-bib-0024] Hatchwell, B. J. (2010). Cryptic kin selection: Kin structure in vertebrate populations and opportunities for kin‐directed cooperation. Ethology, 116, 203–216. 10.1111/j.1439-0310.2009.01732.x

[ece38342-bib-0025] Hatchwell, B. J. , Anderson, C. , Ross, D. J. , Fowlie, M. K. , & Blackwell, P. G. (2001). Social organization of cooperatively breeding long‐tailed tits: Kinship and spatial dynamics. Journal of Animal Ecology, 70, 820–830. 10.1046/j.0021-8790.2001.00541.x

[ece38342-bib-0026] Hinde, A. (2008). The biological significance of the territories of birds. Ibis, 98, 340–369. 10.1111/j.1474-919X.1956.tb01419.x

[ece38342-bib-0027] Humphries, D. J. , Finch, F. M. , Bell, M. B. V. , & Ridley, A. R. (2015). Calling where it counts: Subordinate pied babblers target the audience of their vocal advertisements. PLoS One, 10, e0130795. 10.1371/journal.pone.0130795 26177094 PMC4503734

[ece38342-bib-0028] Jin, L. , Liang, J. , Fan, Q. , Yu, J. , Sun, K. , & Wang, H. (2020). Male Great Tits (*Parus major*) adjust dear enemy effect expression in different breeding stages. Journal of Ornithology, 162, 221–229. 10.1007/s10336-020-01815-3

[ece38342-bib-0029] Kitchen, A. M. , Gese, E. M. , Waits, L. P. , Karki, S. M. , & Schauster, E. R. (2005). Genetic and spatial structure within a swift fox population. Journal of Animal Ecology, 74, 1173–1181. 10.1111/j.1365-2656.2005.01017.x

[ece38342-bib-0030] Leiser, J. K. (2003). When are neighbours ‘dear enemies’ and when are they not? The responses of territorial male variegated pupfish, *Cyprinodon variegatus*, to neighbours, strangers and heterospecifics. Animal Behavior, 65, 453–462. 10.1006/anbe.2003.2087

[ece38342-bib-0031] Low, M. (2006). The energetic cost of mate guarding is correlated with territorial intrusions in the New Zealand stitchbird. Behavioral Ecology, 17, 270–276. 10.1093/beheco/arj025

[ece38342-bib-0032] Mappes, T. , Ylonen, H. , & Viitala, J. (1995). Higher reproductive success among kin groups of bank voles (*Clethrionomys glareolus*). Ecology, 76, 1276–1282. 10.2307/1940934

[ece38342-bib-0033] Mares, R. , Young, A. J. , & Clutton‐Brock, T. H. (2012). Individual contributions to territory defence in a cooperative breeder: Weighing up the benefits and costs. Proceedings of the Royal Society B‐Biological Sciences, 279, 3989–3995. 10.1098/rspb.2012.1071 PMC3427572 22810429

[ece38342-bib-0034] Mazerolle, D. F. , & Hobson, K. A. (2004). Territory size and overlap in male ovenbirds: Contrasting a fragmented and contiguous boreal forest. Canadian Journal of Zoology, 82, 1774–1781. 10.1139/z04-175

[ece38342-bib-0035] Mirville, M. O. , Ridley, A. R. , Samedi, J. P. M. , Vecellio, V. , Ndagijimana, F. , Stoinski, T. S. , & Grueter, C. C. (2018a). Low familiarity and similar ‘group strength’ between opponents increase the intensity of intergroup interactions in mountain gorillas (*Gorilla beringei beringei*). Behavioral Ecology and Sociobiology, 72(11), 1–14. 10.1007/s00265-018-2592-5

[ece38342-bib-0036] Mirville, M. O. , Ridley, A. R. , Samedi, J. P. M. , Vecellio, V. , Ndagijimana, F. , Stoinski, T. S. , & Grueter, C. C. (2018b). Factors influencing individual participation during intergroup interactions in mountain gorillas. Animal Behavior, 144, 75–86. 10.1016/j.anbehav.2018.08.003

[ece38342-bib-0037] Müller, C. A. , & Manser, M. B. (2007). ‘Nasty neighbours’ rather than ‘dear enemies’ in a social carnivore. Proceedings of the Royal Society B‐Biological Sciences, 274, 959–965. 10.1098/rspb.2006.0222 PMC2141673 17251103

[ece38342-bib-0038] Nelson‐Flower, M. J. , Flower, T. P. , & Ridley, A. R. (2018). Sex differences in the drivers of reproductive skew in a cooperative breeder. Molecular Ecology, 27, 2435–2446. 10.1111/mec.14587 29663552

[ece38342-bib-0039] Nelson‐Flower, M. J. , Hockey, P. A. R. , O’Ryan, C. , Raihani, N. J. , du Plessis, M. A. , & Ridley, A. R. (2011). Monogamous dominant pairs monopolize reproduction in the cooperatively breeding pied babbler. Behavioral Ecology, 22, 559–565. 10.1093/beheco/arr018

[ece38342-bib-0040] Nelson‐Flower, M. J. , Hockey, P. A. , O’Ryan, C. , & Ridley, A. R. (2012). Inbreeding avoidance mechanisms: Dispersal dynamics in cooperatively breeding southern pied babblers. Journal of Animal Ecology, 81, 876–883. 10.1111/j.1365-2656.2012.01983.x 22471769

[ece38342-bib-0041] Nelson‐Flower, M. J. , Wiley, E. M. , Flower, T. P. , & Ridley, A. R. (2018). Individual dispersal delays in a cooperative breeder: Ecological constraints, the benefits of philopatry and the social queue for dominance. Journal of Animal Ecology, 87, 1227–1238. 10.1111/1365-2656.12814 29560614

[ece38342-bib-0042] Newey, P. S. , Robson, S. K. A. , & Crozier, R. H. (2010). Weaver ants *Oecophylla smaragdina* encounter nasty neighbors rather than dear enemies. Ecology, 91, 2366–2372. 10.1890/09-0561.1 20836458

[ece38342-bib-0043] R Core Team . (2020). R: A language and environment for statistical computing. R Foundation for Statistical Computing. https://www.R‐project.org/

[ece38342-bib-0044] Radford, A. N. (2005). Group‐specific vocal signatures and neighbour–stranger discrimination in the cooperatively breeding green woodhoopoe. Animal Behavior, 70, 1227–1234. 10.1016/j.anbehav.2005.04.002

[ece38342-bib-0045] Raihani, N. J. , Nelson‐Flower, M. J. , Golabek, K. A. , & Ridley, A. R. (2010). Routes to breeding in cooperatively breeding pied babblers Turdoides bicolor. Journal of Avian Biology, 41, 681–686. 10.1111/j.1600-048X.2010.05211.x

[ece38342-bib-0046] Ridley, A. R. , & Raihani, N. (2007). Facultative response to a kleptoparasite by the cooperatively breeding pied babbler. Behavioral Ecology, 18, 324–330. 10.1093/beheco/arl092

[ece38342-bib-0047] Schielzeth, H. (2010). Simple means to improve the interpretability of regression coefficients. Methods in Ecology and Evolution, 1, 103–113. 10.1111/j.2041-210X.2010.00012.x

[ece38342-bib-0048] Sera, W. E. , & Gaines, M. S. (1994). The effect of relatedness on spacing behavior and fitness of female prairie voles. Ecology, 75, 1560–1566. 10.2307/1939617

[ece38342-bib-0049] Skaug, H. , Fournier, D. , Nielsen, A. , Magnusson, A. , & Bolker, B. (2014). Generalized linear mixed models using AD model builder.

[ece38342-bib-0050] Stamps, J. A. (1984). Growth costs of territorial overlap: Experiments with juvenile lizards (*Anolis aeneus*). Behavioral Ecology and Sociobiology, 15, 115–119. 10.1007/BF00299378

[ece38342-bib-0051] Stamps, J. A. (1990). The effect of contender pressure on territory size and overlap in seasonally territorial species. American Naturalist, 135, 614–632. 10.1086/285065

[ece38342-bib-0052] Stamps, J. A. , & Buechner, M. (1985). The territorial defense hypothesis and the ecology of insular vertebrates. The Quarterly Review of Biology, 60, 155–181. 10.1086/414314 3895283

[ece38342-bib-0053] Støen, O.‐G. , Bellemain, E. , Sæbø, S. , & Swenson, J. E. (2005). Kin‐related spatial structure in brown bears Ursus arctos. Behavioral Ecology and Sociobiology, 59, 191–197. 10.1007/s00265-005-0024-9

[ece38342-bib-0054] Temeles, E. J. (1994). The role of neighbours in territorial systems: When are they “dear enemies”? Animal Behavior, 47, 339–350. 10.1006/anbe.1994.1047

[ece38342-bib-0055] Thompson, F. J. , Marshall, H. H. , Vitikainen, E. I. K. , & Cant, M. A. (2017). Causes and consequences of intergroup conflict in cooperative banded mongooses. Animal Behavior, 126, 31–40. 10.1016/j.anbehav.2017.01.017

[ece38342-bib-0056] Uy, F. M. K. , Adcock, J. D. , Jeffries, S. F. , & Pepere, E. (2019). Intercolony distance predicts the decision to rescue or attack conspecifics in weaver ants. Insectes Sociaux, 66, 185–192. 10.1007/s00040-018-0674-z

[ece38342-bib-0057] Van Belle, S. , & Estrada, A. (2020). The influence of loud calls on intergroup spacing mechanism in black howler monkeys (*Alouatta pigra*). International Journal of Primatology, 41, 265–286. 10.1007/s10764-019-00121-x

[ece38342-bib-0058] Walker, F. M. , Taylor, A. C. , & Sunnucks, P. (2008). Female dispersal and male kinship–based association in southern hairy‐nosed wombats (*Lasiorhinus latifrons*). Molecular Ecology, 17, 1361–1374. 10.1111/j.1365-294X.2008.03670.x 18302694

[ece38342-bib-0059] Wiley, E. M. , & Ridley, A. R. (2016). The effects of temperature on offspring provisioning in a cooperative breeder. Animal Behavior, 117, 187–195. 10.1016/j.anbehav.2016.05.009

[ece38342-bib-0060] Zack, S. (2010). Coupling delayed breeding with short‐distance dispersal in cooperatively breeding birds. Ethology, 86, 265–286. 10.1111/j.1439-0310.1990.tb00435.x

